# Treadmill exercise stress cardiac MRI for the assessment of left ventricular wall motion: a comparison with stress echocardiography in healthy volunteers

**DOI:** 10.1186/1532-429X-14-S1-O35

**Published:** 2012-02-01

**Authors:** Paaladinesh Thavendiranathan, Jennifer Dickerson, Debbie Scandling, Vijay Balasubramanian, Michael Pennell, Alice M Hinton, Subha V Raman, Orlando P Simonetti

**Affiliations:** 1 Cardiovascular Medicine, Cleveland Clinic Foundation, Cleveland, OH, USA; 2 Cardiovascular Medicine, The Ohio State University, Columbus, OH, USA

## Background

Although exercise stress echo is commonly used for ischemic assessment, acoustic window limitations can often affect diagnosis. Recently, treadmill exercise stress CMR has become feasible (Foster et al, MRM, 2011); however, a direct comparison with stress echo has not been performed. The objective of this study was to compare exercise stress CMR to echo in healthy volunteers to assess left ventricular wall motion at peak stress.

## Methods

28 volunteers (aged 28 ± 11 years, 15 males) underwent both exercise stress echo and CMR. They were randomly assigned to one modality first and underwent the second modality within 1 week. CMR was performed using an MRI-compatible treadmill adjacent to the MRI table. Resting cine images (5 short axis, 3 long axis) were acquired first. Patients subsequently exercised on the treadmill (Bruce protocol) and at peak exercise returned to the magnet for stress imaging. Treadmill exercise stress echo was performed as per routine protocol. Timing to begin imaging, patient hemodynamics, and exercise parameters were recorded. A blinded reviewer (>5 years experience reading stress echo and CMR) independently reviewed CMR and echo images for adequate visualization of all 17 myocardial segments and image quality for wall motion analysis (Figure). Endocardial visualization index was calculated for each patient as number of evaluable segments/total number of segments.

**Figure 1:**
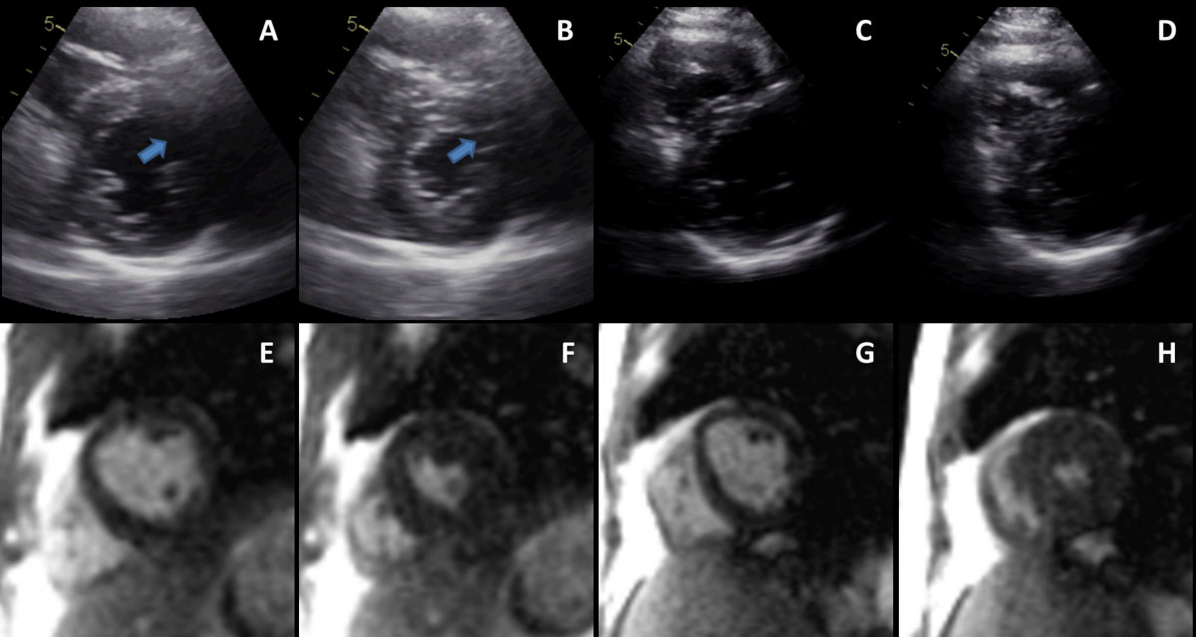
Illustration of echocardiography and CMR images from one volunteer. Panels A and B are rest diastolic and systolic frames. The anterior lateral wall was not adequately seen both in systole and diastole but is clearly seen in the corresponding CMR slice positions (Panels E and F). At peak stress (Panels C and D) the endocardial walls by echocardiography were difficult to assess in multiple segments while all segments were clearly seen on CMR images (Panels G and H). Heart rate at time of peak images was 162 for echocardiography and 155 for CMR.

## Results

Timing, hemodynamic, and exercise parameters are summarized in the Table. HR at the time of image acquisition was similar between the studies. Average time from cessation of exercise to image acquisition (21 vs. 31 seconds) and time to complete imaging (41 vs. 114 seconds) was shorter for stress CMR . At rest, all 17 myocardial segments were adequately visualized in 27/28 CMR studies and 17/28 echo studies (p=0.002, McNemar’s test). At peak stress, all 17 myocardial segments were visualized in 27/28 CMR studies, and 17/28 echo studies (p=0.002, McNemar's test). The median (range) number or segments inadequately visualized at rest and stress by echo were 1 (1-4) and 2 (1-6), respectively, while by CMR was 1 segment at both rest and stress. The mean±SD endocardial visualization index at stress for echo and CMR were 0.94±0.10 and 1.0±0.01, respectively (p=0.004, paired t-test).

**Table 1:** 

	Stress echocardiography	Stress CMR	p Value*
Resting Variables			

HR, bpm	73 ± 12	74 ± 13	0.7
Systolic BP, mmHg	117 ± 16	111 ± 13	0.05
Diastolic BP, mmHg	71 ± 8	70 ± 9	0.5

Exercise Variables			

Exercise duration, mins	11.4 ± 2.1	11.1 ± 2.2	0.07
Peak HR, bpm	177 ± 11	173 ± 10	0.01
Peak systolic BP, mmHg	155 ± 19	158 ± 19	0.3
Peak diastolic BP, mmHg	70 ± 9	76 ± 9	0.003
Time to start stress imaging, seconds	31 ± 7	21 ± 2	<0.001
HR at time of peak cine images, bpm	150 ± 16	148 ± 14	0.7
Time to complete stress imaging, seconds	114 ± 23	41 ± 2	<0.001

*paired t test; HR, heart rate; bpm, beats per minute; BP, blood pressure; mmHg, millimeters of mercury; min, minutes; CMR, cardiac magnetic resonance imaging.

## Conclusions

Exercise stress CMR to assess peak exercise wall motion is feasible and can be performed as rapidly as stress echo. Stress CMR was superior to echo in adequately assessing all myocardial segments both at rest and peak stress. The potential superiority of stress CMR for ischemia identification needs to be formally evaluated.

## Funding

National Institute of Health R01.

